# Impact of Different Body Positions on Bioelectrical Activity of the Pelvic Floor Muscles in Nulliparous Continent Women

**DOI:** 10.1155/2015/905897

**Published:** 2015-02-22

**Authors:** Daria Chmielewska, Magdalena Stania, Grzegorz Sobota, Krystyna Kwaśna, Edward Błaszczak, Jakub Taradaj, Grzegorz Juras

**Affiliations:** ^1^Department of Physiotherapy Basics, Jerzy Kukuczka Academy of Physical Education, Mikołowska 72a, 40-065 Katowice, Poland; ^2^Department of Human Motor Behavior, Jerzy Kukuczka Academy of Physical Education, Mikołowska 72a, 40-065 Katowice, Poland; ^3^Department and Faculty of Medical Biophysics, Medical University of Silesia, 18 Medyków Street, 40-752 Katowice, Poland

## Abstract

We examined pelvic floor muscles (PFM) activity (%MVC) in twenty nulliparous women by body position during exercise as well as the activation of abdominal muscles and the gluteus maximus during voluntary contractions of the PFMs. Pelvic floor muscle activity was recorded using a vaginal probe during five experimental trials. Activation of transversus abdominis, rectus abdominis, and gluteus maximus during voluntary PFM contractions was also assessed. Significant differences in mean normalized amplitudes of baseline PFM activity were revealed between standing and lying (*P* < 0.00024) and lying and ball-sitting positions (*P* < 0.0053). Average peak, average time before peak, and average time after peak did not differ significantly during the voluntary contractions of the PFMs. Baseline PFM activity seemed to depend on the body position and was the highest in standing. Pelvic floor muscles activity during voluntary contractions did not differ by position in continent women. Statistically significant differences between the supine lying and sitting positions were only observed during a sustained 60-second contraction of the PFMs.

## 1. Introduction

Surface electromyography (sEMG) is among the modalities to investigate the function of pelvic floor muscles in real time [1]. Electromyography records and quantifies the electrical activity generated by muscle fibres; depolarisation and repolarization of the surface membrane of muscle fibres are the source of the electrical potential changes detected. Experts recommend sEMG as a method of the evaluation of pelvic floor muscles (PFMs) function [2].

Surface electromyography (sEMG), as a noninvasive method, was used to compare pelvic floor muscle activity in incontinent patients and asymptomatic women [3]. It has also been applied to investigate activation levels of the abdominal muscles as well as the patterns of pelvic floor muscle activity in continent women and to determine whether these patterns are maintained or altered in incontinent individuals [4, 5]. It was shown that, in healthy individuals, activation of the transversus abdominis was a physiological response to an increase in pelvic floor muscle contraction [6]. Simultaneous contractions of the pelvic floor and transversus abdominis [6, 7] have been accounted for by several anatomical and biochemical characteristics they have in common [5]. MRI-based investigations of Soljanik et al. [7] demonstrated synchronous movement of the fossa ischioanalis, levator ani, and gluteus maximus. Coactivation of the pelvic floor and surrounding muscles can be associated with body and lumbopelvic posture [8] as well as sitting posture [9] which seems important while working with incontinent patients. Urine usually leaks out in the standing position that resulted from gravity and pressure from the pelvic organs on the musculofascial supportive structures. Patients with urinary incontinence and/or pelvic pain should be instructed with respect to body positions assumed during exercise and everyday routines. Several authors investigated the relationship between body position and resting/voluntary PFM activation. Electromyographic activity was observed during three rapid contractions (5 s) in supine and standing positions. Incontinent women had lower PFM activity in standing compared to healthy controls. The researchers also noted that the EMG activity of pelvic floor muscles decreased with age [3].

Incontinent subjects of Capson et al. [8] exhibited significantly higher resting PFM activity in all postures in standing position compared to supine position. Resting PFM activity was higher in the standing hyperlordotic posture as compared to the normal and hyperlordotic postures. The analysis showed that incontinent women also had higher intravaginal pressure in standing than in the supine position. The maximal intravaginal pressure and the time of sustained maximal voluntary contraction did not differ significantly by position [10].

Other researchers did not observe differences between the levels of PFM activation generated by voluntary PFM contractions in the standing and supine positions in continent women. However, they did find differences in muscle activation sequence by position [11]. Auchincloss and McLean [12] demonstrated between-trial reliability of EMG data recorded from the PFMs using two different vaginal probes with the subjects performing maximum voluntary contractions and a coughing task in two positions: supine and standing. Due to differences in the results regarding the impact of body position on the resting and voluntary PFM activity, it was hypothesized that the position might alter the level of the activity and contraction velocity. It might also affect the activity of the abdominal and gluteal muscles.

In this study, we investigated bioelectrical activity of the pelvic floor muscles (PFMs) during a voluntary contraction as well as the activity of the abdominal muscles and the gluteus maximus in three positions: supine lying, standing, and sitting on the ball (from now on referred to as* sitting*), that is, body positions typically assumed during daily routines.

Similar to other researchers [11, 13], we decided to recruit young asymptomatic women to eliminate the effects of neurological disorders or PFM weakness. Also, a document issued by the members of the International Continence Society (ICS) points out the need to define normal values for pelvic floor muscles function when measured with sEMG [2]. Reference ranges would undoubtedly facilitate a comparison of therapy outcomes between individuals, especially between women with PFM dysfunction, which additionally justifies carrying out investigations in continent women.

## 2. Materials and Methods

### 2.1. Setting and Participants

**Figure 1:**
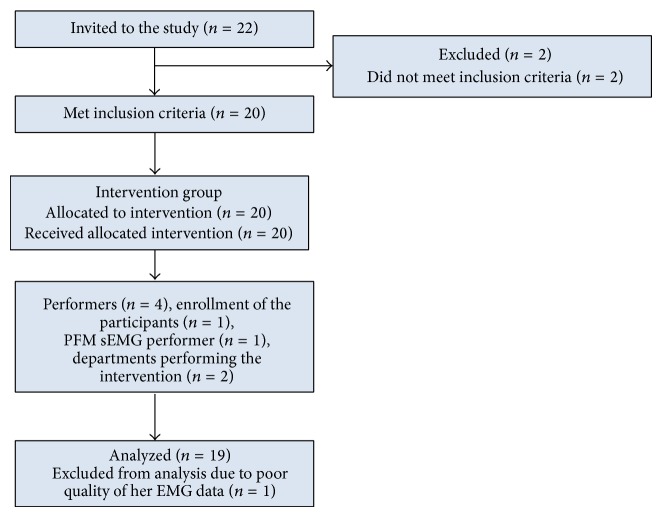
Diagram flow.

Twenty-two continent women aged 19–28 were invited to participate. Two of them were excluded from the group since they did not meet the inclusion criteria, and so ultimately 20 healthy nulliparous women entered the study (Figure 1).

Exclusion criteria included a history of SUI, pregnancy, childbirth(s), pelvic surgery, diabetes, hypertension, neurological abnormalities, urinary tract infection, elevated temperature, practicing professional sport, spinal pain, and body mass index over 30 kg/m^2^. Candidates were supplied with a comprehensive description of the aim and methods of the study. After obtaining their informed consent, a personal history was taken from each participant. Demographic data included age, height, weight, body mass index, and employment status.

### 2.2. Randomization and Interventions

The measurements were taken in late morning hours to minimize the impact of fatigue. The subjects were asked not to take up intensive physical exercises 24 hours before the measurement. Temperature in the examination room was 24°C. The study protocol was approved by the Bioethics Committee. The measurements were performed under standard testing conditions similarly for all subjects.

Pelvic floor sEMG activity was recorded using a small diameter vaginal probe with two metal sensors (Everyway Medical Instruments Co). The probe was inserted using a small amount of antiallergic lubricant with the sensors positioned laterally in the vagina. Vaginal electrode placement was checked during breaks between the consecutive measurement sessions. After cleansing the skin site with an alcohol swab, round self-adhesive electrodes (silver/silver chloride) were applied to the skin over the examined muscle (surface electromyography for the noninvasive assessment of muscles) [14].

The reference surface electrode was placed over the right anterior superior iliac spine (ASIS). The next two bipolar self-adhesive electrodes were located on the right side along muscle fibres of the rectus abdominis, transversus abdominis, and gluteus maximus. Two sEMG sensors were placed at the following locations:

1. rectus abdominis (RA), 1.5 cm lateral and caudal to the umbilicus;
2. transversus abdominis (TA), 2 cm cephalic to the pubic bone and parallel to the superior pubic ramus;
3. gluteus maximus (GM) at 50% on the line between the sacral vertebrae and the greater trochanter.

The ability to contract the rectus abdominis and transversus abdominis without moving the pelvis was assessed prior to sensor placement. Prior to measurements, the participants were asked to urinate a full void. All subjects were instructed on the correct contraction of pelvic floor muscles and could observe sEMG signals on the computer monitor during the instruction session. All sEMG recordings were performed by the same investigator.

### 2.3. Testing Procedure

The experiment consisted of two phases: (1) the MVC procedure to recruit each of the examined muscles and (2) five trials designed to determine pelvic floor muscle activity, that is, a 10-second baseline activity, 5 repeated short (quick flick) contractions, 5 repetitions of 10-second voluntary contractions, sustained 60-second contraction, and 10-second relaxation. During the first phase, each participant was instructed to perform maximal voluntary contractions (MVCs) of each examined muscle separately and as forcefully as possible for about 5 seconds. During MVCs verbal encouragement was provided. Three attempts were made with 60-second rests between each contraction to reduce the effect of muscle fatigue. The order of muscle MVC testing was randomly assigned. MVCs were used as reference values for each muscle group [15].

*The MVC Procedure to Recruit Pelvic Floor Muscles*. Supine lying: the hip and knee were positioned at 30° and 90° of flexion, respectively. The positions were controlled with the goniometer. Using a wedge under the feet, the ankles were positioned at 0° of flexion since 10° dorsiflexion promotes the activity of pelvic floor muscles [16]. No visible contractions of the rectus abdominis or gluteus muscles were allowed.

*The MVC Procedure to Recruit the Rectus Abdominis*. This procedure included the following: feet flat secured to the table with a strap, supported sitting in a 30-degree position, the subject's shoulder girdle strapped to the table, and a sit-up attempt.

*The MVC Procedure to Recruit the Transversus Abdominis*. This procedure included supine lying with abdominal hollowing exercises [17].

*The MVC Procedure to Recruit the Gluteus Maximus*. This procedure included the following: prone lying, pelvic girdle strapped to the table, overextension in the hip joint, and lower leg secured to the table with the knee in full extension.

Following the MVC procedure, sEMG signal was recorded during each trial [18]:

- a 10-second baseline sEMG recording (ability to relax the muscles) (parameters measured were mean amplitude (%MVC));
- 5 repeated short (quick flick) contractions with a 5-second pause between each contraction (parameters measured were average peak (%MVC) (average value of all five local peaks, calculated when the amplitude exceeds the threshold level predetermined as 50% between minimum and maximum amplitudes in each particular trial), average mean (%MVC) (mean amplitude value of the active sEMG portions, calculated when the amplitude exceeds the threshold level predetermined as 50% between minimum and maximum amplitudes in each particular trial), average time before peak (s), and the average duration needed to let the signal increase to the local peak);
- 5 repetitions of 10-second voluntary contractions with 10 seconds of rest in-between (parameters measured were average peak (%MVC) (average value of all local peaks detected, calculated when the amplitude exceeds the threshold level predetermined as 50% between minimum and maximum amplitudes in each particular trial) and average mean (%MVC) (mean amplitude value of the active EMG portions, calculated when the amplitude exceeds the threshold level predetermined as 50% between minimum and maximum amplitudes in each particular trial));
- a sustained 60-second contraction (parameters measured were average mean (%MVC) (mean amplitude value of the active sEMG portions, calculated when the amplitude exceeds the threshold level predetermined as 50% between minimum and maximum amplitudes in each particular trial)).
- The following instruction was given to participants: “Pull up and in and squeeze around the probe as strongly as you can until you hear the command* Now relax.*” Verbal feedback was given during the trial;
- a 10-s relaxation (Resting tone) immediately after the 60-s contraction (parameters measured: were mean amplitude (%MVC)).

### 2.4. Instrumentation

Pelvic floor muscles sEMG was recorded using Myo Trace 400 (Noraxon U.S.A. Inc.) with a preamplifier (band pass filter 20 Hz–500 Hz, common mode rejection ratio of >100 dB at 60 Hz, input impedance >100 MΩ, and amplifier gain 500). A 16-bit analog to digital (A/D) converter with an antialiasing filter set to 500 Hz frequency was also used.

### 2.5. Signal Processing

The raw sEMG data were full wave rectified. Root mean square (RMS) values were calculated using a 100 ms sliding window.

Surface EMG amplitude data are strongly influenced by detection conditions. One solution of this problem is the normalization of the sEMG signal to a reference value. The most common method is referred to as MVC-normalization, referring to a maximum voluntary contraction performed for each muscle prior to the test trials. The sEMG level is then expressed as %MVC [19]. Amplitude sEMG signals of the pelvic floor muscles in standing and sitting were normalized to the MVC performed in the supine-lying position (see above for positions of the MVC test).

### 2.6. Statistical Analysis

Friedman's two-way ANOVA for ranks is used when the same parameter has been measured several times (*k* ≥ 2) under different conditions on the same subjects. The Tukey's post hoc test, which reveals which means are significantly different from each other, was also performed. The significance level was set at (*P* < 0.05).

## 3. Results

Our study group was homogeneous regarding age; body height, weight, and body mass index; and employment status. Mean age was 23.6 years (SD 1.13) and mean BMI was 20.3 kg/m^2^ (SD 1.9). Electromyographic recordings of one person were excluded from analysis due to artifact signals.

Body position had little effect on the activity of the gluteus maximus; therefore, only the baseline, sustained 60-second contraction and resting tone data are presented.

### 3.1. Baseline sEMG Recording

**Figure 2:**
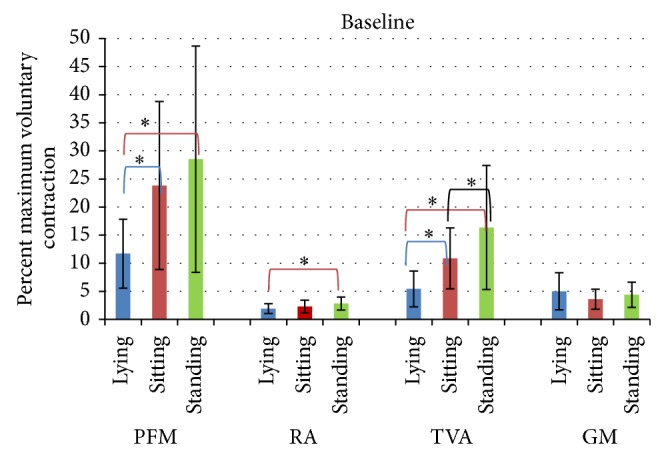
Normalized amplitude (%MVC) for rectus abdominis (RA) and transversus abdominis (TVA) and gluteus maximus (GM) in lying, sitting, and standing position during the baseline sEMG recording of pelvic floor muscles.

The analysis of the baseline test results revealed statistically significant differences between supine lying, standing, and sitting. The mean resting activity of PFMs was the lowest in supine lying (Figure 2) and was significantly different compared to standing (*P* = 0.00024) and sitting (*P* = 0.0053) positions. No statistically significant differences were seen between standing and sitting positions (*P* = 0.4).

The mean normalized resting activity of the rectus abdominis in standing was significantly higher compared to supine lying *P* < 0.0026.

An increase in the activity of the transversus abdominis was observed in standing. The mean normalized resting sEMG amplitude of the transversus abdominis activity differed significantly between all study positions, supine lying versus sitting (*P* = 0.0037), supine lying versus standing (*P* = 0.00012), and standing versus sitting (*P* = 0.0036).

The mean amplitude of the sEMG signal from the gluteus maximus was the highest in supine lying; however, its value did not differ significantly from those noted in sitting and standing.

### 3.2. Short (Quick Flick) Contractions

Normalized average mean of the sEMG amplitude was significantly different between lying and standing (Table 1). Average peak (Table 1) and average time before peak (Table 2) do not differ significantly during the voluntary contractions of the PFMs.

The parameters measured for the rectus abdominis did not differ by position (Tables 1 and 2).

The transversus abdominis actively responded to voluntary contractions of the PFMs. Average time before peak was not significantly different in all three positions (Table 2). Average peak (%MVC) differed significantly between supine lying versus sitting, standing, and sitting. Average mean (%MVC) differed significantly between supine lying and sitting (Table 1).

### 3.3. 5 Repetitions of 10-Second Voluntary Contractions with 10 Seconds of Rest In-Between

No differences were found between mean sEMG amplitudes (normalized to the MVC) and average peak (normalized to the MVC) of the pelvic floor muscles in all study positions. The activity of the PFMs was approximately 80% and 98% of the MVC in supine lying and standing, respectively (Table 3).

Electromyography signals from the abdominal muscles recorded during the voluntary contraction of the PFMs differed by position. The activity of the rectus abdominis in standing was greater compared to supine lying (Table 3). Mean amplitude (%MVC) and average peak of the transversus abdominis amplitude (%MVC) differed significantly between standing and sitting. The differences reached statistical significance (the parameters exhibited the highest values in standing) (Table 3). Supine lying and sitting differed significantly only regarding normalized mean amplitude.

### 3.4. Sustained 60-Second Contraction

Significant differences in mean normalized amplitudes of baseline PFM activity were revealed between standing and lying (*P* < 0.00024) and lying and ball-sitting positions (*P* < 0.0053).

Significant differences in mean normalized sEMG amplitudes during a sustained 60-second contraction were revealed between lying and sitting. The greatest and the smallest electromyographic activities from the PFM were observed in the sitting and lying positions, respectively (Table 4).

Electromyography signals from the abdominal muscles recorded during a sustained 60-second contraction of the PFMs differed by position (Table 4). Mean amplitude of the transversus abdominis (%MVC) differed significantly between lying and sitting and standing and sitting both during the 60-second contraction and 5 repetitions of 10-second voluntary PFM contraction. The highest amplitudes were observed in the standing position (Tables 3 and 4).

The activity of the gluteus maximus differed by position with the highest amplitude in sitting (Table 4).

### 3.5. Resting Tone

**Figure 3:**
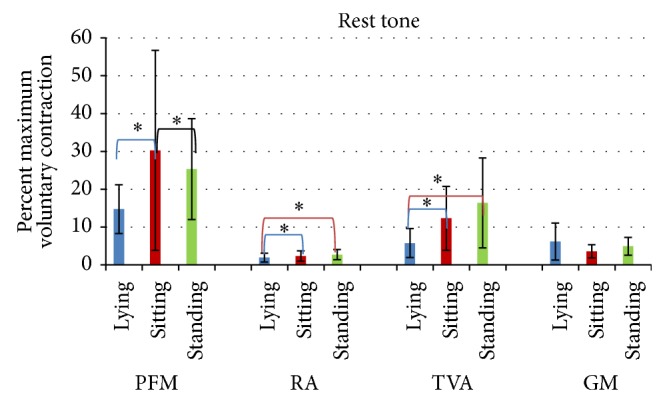
Normalized amplitude (%MVC) for rectus abdominis and transversus abdominis in lying, sitting, and standing position during the 10-second relaxation test for pelvic floor muscles.

Following a 60-second contraction, pelvic floor muscles were capable of relaxation as demonstrated by low sEMG amplitude. Normalized mean amplitude of the sEMG signal was the lowest in supine lying (not significantly lower compared to the standing position) (Figure 3).

The activity of the rectus abdominis differed significantly by position. The lowest amplitude (%MVC) was noted in supine lying; its value was significantly lower than in standing (*P* = 0.00014) and sitting (*P* = 0.017).

The mean sEMG signal amplitude of the transversus abdominis was also effectively lowered. The lowest amplitude (%MVC) was noted in supine lying (significantly lower than in standing (*P* = 0.0002) and sitting (*P* = 0.014)).

The activity of the gluteus maximus did not differ significantly by position.

## 4. Discussion

Pelvic floor musculature constitutes a specific group of striated muscles. Contrary to the reciprocal innervation of limb muscles, pelvic floor muscles are characterized by synchronous and harmonic contractions [20] and prolonged tension (except for micturition and defecation) [21]. Electromyographic examinations demonstrate that pelvic floor muscles exhibit slight but continuous motor unit potential activation evenat rest. The continuous firing of motor units in the PFMs is related to an appropriate excitatory set-up of their motor neurons derived from suprasegmental and segmental inputs [20].

This resting potential is a result of the activity of slow twitch muscle fibres [3] which predominate among the components of deep pelvic floor muscles [12]. Resting PFM activity also seems to depend on changes in lumbopelvic posture [8].

The lowest resting activity of the PFMs, suggesting only slight motor unit potential activation, was observed in supine lying with the knees flexed compared to standing (*P* = 0.00024) and sitting (*P* = 0.0053). The highest mean sEMG amplitude (%MVC) was obtained during standing. It has been suggested that, due to gravitational forces, the pressure on the structures of the abdomen and lesser pelvis increases in the standing position. Increased pressure on the urinary bladder and urethra increases, in turn, pelvic floor muscle tone [11]. In supine lying, the gravitational force mainly affects the posterior wall of the abdominal cavity and not the floor of the lesser pelvis. Capson et al. [8] also observed significantly lower resting values of PFMs' activity in supine lying compared to standing. Other authors did not find any significant effects of pelvic orientation on PFM activity at rest and during voluntary contraction [22].

Resting recordings obtained from the PFMs, abdominal muscles, and gluteus maximus revealed that the activity of the transversus abdominis was greater in standing compared to supine lying (*P* = 0.00012) and sitting (*P* = 0.0036). The activity of the rectus abdominis in standing was greater than in supine lying (*P* = 0.0031), which might have been a result of abdominal pressure changes and gravitational forces.

A 10-second measurement is sufficient to determine the resting sEMG activity of the pelvic floor muscles; the same measurement period was used in other investigations [21].

Strength and endurance are not the only essential characteristics of the PFMs; the rate of PFM activation is also important. Madill et al. [4] observed that the relative time to peak was shorter in incontinent women. Contractile properties of muscle fibres are determined by alpha-motor neurons which may differ in function and morphology. High intensity short-burst contractions engage fast-twitch fibers innervated by phasic motor neurons. The superficial layer of pelvic floor muscles, which reacts to short and rapid increases in intra-abdominal pressure, contains a large number of fast-twitch fibres [23].

Our investigations did not reveal significant differences between mean values of average peak sEMG of the PFMs by study positions. The latter also did not affect average time before peak.

Our results show that young nulliparous women can voluntarily contract their pelvic floor muscles obtaining similar sEMG activities in all positions. PFM activation during five 10-second contractions did not vary by exercise position. Similar conclusions were reached by Madill and McLean [11], who examined the effect of body position on pelvic floor and abdominal (rectus abdominis, external oblique, internal obliques, and transversus abdominis) muscles activation in women aged 21–60 with no history of stress urinary incontinence. The authors found equal sEMG amplitudes in all positions and suggested that the effect of gravity had little to no impact on the PFM activation capacity and functional strength. However, Sapsford et al. [9] demonstrated a relationship between different sitting postures and the levels of pelvic floor and abdominal muscle activity. They also found that pelvic floor muscle activity was the greatest in very tall unsupported sitting.

The present investigations did reveal significant differences in the activity of the PFM during a sustained 60-second contraction, but only between the lying and sitting positions (*P* = 0.0011). A long-lasting contraction during unsupported sitting seems more demanding for the sensorimotor control system compared to lying. It might be that the maintenance of dynamic balance and lumbar spine stability during a voluntary contraction of the PMF significantly improved the activity of these muscles. Burti et al. [24] reported that incontinent women showed worse performance during a 60-second sustained contraction compared to their continent counterparts. Also, sEMG signal amplitude was lower in incontinent women compared to their continent counterparts; however, the magnitude of these differences was not specified.

The lack of statistically significant differences between sEMG amplitudes normalized to %MVC might be associated with young age of our study participants and high activation capacity of their pelvic floor muscles. Incontinent women had lower PFM activities, especially in the standing position, related to age and vaginal deliveries [3]. PFM function is affected by age, the number of vaginal deliveries, BMI, and intense physical exertion [25].

Coactivation of the transversus abdominis and the PFMs was also observed by other researchers. Neumann and Gill [26] found that their continent subjects were unable to contract the pelvic floor effectively, while maintaining relaxation of the transversus abdominis and internal oblique muscles. Synergistic recruitment of the pelvic floor and the transversus abdominis muscles [1, 6, 8, 11] has been accounted for by their common anatomical and biomechanical properties [5]. The fibres of the transversus abdominis are continued into the transverse perineal muscle [5]. In healthy individuals, transversus abdominis activation is a physiological consequence of an increase in PFM tension. Pelvic floor muscles also stabilize the lumbar spine [6] which is essential in standing. Increased activity of the PFMs in the antigravitational position is believed to improve spinal stability.

There have been long-lasting discussions on how to use the synergistic activation of the PFMs and transversus abdominis in women with urinary incontinence. Stüpp et al. [27] confirmed coactivation of the PFMs and transversus abdominis in a population of nulliparous young women. The addition of PFM contraction to the abdominal hypopressive technique significantly increased the amount of transversus abdominis activation.

A comparison of changes in sEMG amplitude with the results reported by other authors poses problems since the majority of those results had not been normalized to MVC [3, 8, 21].

Madill and McLean [11] normalized sEMG results to the maximum voluntary electrical activity attained by each muscle during each contraction; they did not find differences in muscle activation levels by position (lying, standing, and sitting). The activities of the rectus abdominis, external obliques, internal obliques, and transversus abdominis were recorded during voluntary contractions of the PFMs. High activation levels of the transversus abdominis and internal obliques seem to suggest that synergistic coactivation between these muscles and the PFMs is stronger compared to that between the rectus abdominis, external obliques, and the PFMs. The coactivation pattern of the PFMs and abdominal muscles was different in sitting compared to standing and lying. In the sitting position, the rectus abdominis and external oblique muscles do not actively help maintain postural stability. Kinesiological EMG and perineal ultrasound demonstrated an increase in rectus abdominis activity (a two-fold amplitude increase compared to baseline activity) while contracting the pelvic floor muscles in 2 out of 10 nulliparous volunteers [28]. Perineal ultrasound used by Peschers et al. [28] did not demonstrate bladder neck descent during several fatiguing tasks performed in the supine position by continent nulliparous volunteers.

It was hypothesized that postural functions of the PFMs could play a role in voluntary contractions and delayed relaxation [29]. The study of Hove et al. [30] revealed that only 51.3% of a general female population was capable of conscious PFM relaxation after voluntary contraction of the muscles. The ability of our young participants to relax the PFMs after a 60-second contraction was satisfactory. We believe that the activity and relaxation of the PFMs at rest are important when considering voluntary regulation of PFM activity.

Our study has several limitations including a relatively small number of study participants. Also, we could not compare the results with incontinent women as no comparison group had been formed.

Our study group comprised 20 women; the number was not high but some other study groups were even smaller in size [6, 11, 28].

## 5. Conclusion

The following conclusions can be drawn from the present study.

- Resting activity of the PFMs differed significantly by position; the greatest activity was noted in standing.
- Average time to peak and activity levels of PFMs did not differ significantly by position.
- The test position only slightly affected PFM activity during voluntary contractions; a difference was only shown regarding the lying versus standing positions and lying versus sitting positions during quick flick and a sustained 60-second contractions, respectively.
- Abdominal muscles activity was significantly greater in the standing position.

The effect of body position on the activity of the PFMs and synergistic muscles is of considerable importance with respect to women's physical activity as well as the prevention of stress urinary incontinence. Our investigations carried out in a group of young continent women have demonstrated limited influence of body positions on the activity of PFMs.

Specialized sEMG studies are needed to further elucidate the effect of body position on PFMs' activity and to investigate muscle fatigue in continent and incontinent women.

## Figures and Tables

**Table 1:** Normalized amplitude (%MVC) for rectus abdominis and transversus abdominis in lying, sitting, and standing positions during 5 repeated short (quick flick) voluntary pelvic floor muscles contractions.

		Muscles					
Position	*N*	Pelvic floor muscle		Rectus abdominis		Transversus abdominis	
		Mean (%)	SD	Mean (%)	SD	Mean (%)	SD
Average mean amplitude (%MVC)							
Lying	19	62.19	33.23	2.52	1.91	37.7	26.07
Sitting	19	53.03	28.73	2.39	1.62	16.82	10.95
Standing	19	46.27	39.3	3.35	2.55	27.51	19.95
*P* ^*^ (lying/standing/sitting)		**P** < 0.02146		**P** < 0.01832		*P* < 0.00075	
*P* (lying/sitting)		0.16		0.99		**0.0074**	
*P* (lying/standing)		**0.0064**		0.6		0.26	
*P* (standing/sitting)		0.35		0.51		0.23	

Average peak amplitude (%MVC)							
Lying	19	111.35	48.23	4.45	3.23	70.06	48.56
Sitting	19	114.46	69.29	4.69	3.17	38.91	25.81
Standing	19	120.23	99.74	7.13	5.35	65.69	53.14
*P* ^*^ (lying/standing/sitting)		*P* < 0.43081		*P* < 0.19883		*P* < 0.01714	
*P* (lying/sitting)		0.97		0.99		**0.018**	
*P* (lying/standing)		0.81		0.35		0.91	
*P* (standing/sitting)		0.91		0.42		**0.046**	

*P* ^*^: Friedman's ANOVA.

*P*: Tukey's post hoc test.

**Table 2:** Average time before peak of the rectus abdominis and transversus abdominis in lying, sitting, and standing during 5 repeated short (quick flick) voluntary pelvic floor muscles contractions.

		Muscles					
Position	*n*	Pelvic floor muscles		Rectus abdominis		Transversus abdominis	
		Mean (s)	SD	Mean (s)	SD	Mean (s)	SD
Average time before peak (s)							
Lying	19	0.39	0.18	0.32	0.18	0.32	0.15
Sitting	19	0.3	0.12	0.19	0.11	0.33	0.24
Standing	19	0.36	0.13	0.28	0.09	0.3	0.16
*P* ^*^ (lying/standing/sitting)		*P* < 0.85394		*P* < 0.19883		*P* < 0.12662	
*P* (lying/sitting)		0.1		0.088		0.99	
*P* (lying/standing)		0.71		0.76		0.93	
*P* (standing/sitting)		0.4		0.3		0.91	

*P* ^*^: Friedman's ANOVA.

*P*: Tukey's post hoc test.

**Table 3:** Normalized amplitude (%MVC) for rectus abdominis and transversus abdominis in lying, sitting, and standing positions during 5 repetitions of 10-second voluntary pelvic floor muscles contractions.

		Muscles					
Position	*n*	Pelvic floor muscle		Rectus abdominis		Transverses abdominis	
		Mean (%)	SD	Mean (%)	SD	Mean (%)	SD
Average mean amplitude (%MVC)							
Lying	19	80.07	30.7	3.82	1.92	53.45	29.98
Sitting	19	82.19	40.52	5.23	3.47	35.25	19.47
Standing	19	98.28	62.66	7.49	5.07	61.11	38.94
*P* ^*^ (lying/standing/sitting)		*P* < 0.85394		*P* < 0.11605		**P** < 0.01951	
*P* (lying/sitting)		0.98		0.56		**0.077**	
*P* (lying/standing)		0.21		**0.031**		0.61	
*P* (standing/sitting)		0.29		0.23		**0.0086**	

Average peak amplitude (%MVC)							
Lying	19	108.83	41.99	5.16	3.15	72.48	39.95
Sitting	19	110.93	60.74	6.97	4.23	45.93	27.58
Standing	19	129.45	89.75	9.96	7.16	80.5	53.48
*P* ^*^ (lying/standing/sitting)		*P* < 0.81016		**P** < 0.00728		**P** < 0.01951	
*P* (lying/sitting)		0.99		0.6		0.058	
*P* (lying/standing)		0.29		**0.043**		0.75	
*P* (standing/sitting)		0.37		0.26		**0.011**	

*P* ^*^: Friedman's ANOVA.

*P*: Tukey's post hoc test.

**Table 4:** Normalized amplitude (%MVC) for rectus abdominis, transversus abdominis, and gluteus maximus in lying, sitting, and standing positions during a sustained 60-second voluntary pelvic floor muscles contraction.

		Muscles							
Position	*N*	Pelvic floor muscle		Rectus abdominis		Transversus abdominis		Gluteus maximus	
		Mean (%)	SD	Mean (%)	SD	Mean (%)	SD	Mean (%)	SD
Mean amplitude (%MVC)									
Lying	19	60.87	14.25	3.88	2.1	43.88	20.97	6.25	3.8
Sitting	19	63.5	18.55	4.07	3.02	31.43	17.44	8.62	5.9
Standing	19	62.23	21.65	4.73	3.18	44.02	21.2	7.84	4.05
*P* ^*^ (lying/standing/sitting)		**P** < 0.000001		**P** < 0.000001		**P** < 0.000001		**P** < 0.00002	
*P* (lying/sitting)		**0.0011**		**0.041**		**0.000022**		**0.000021**	
*P* (lying/standing)		0.2		**0.000022**		0.98		**0.000025**	
*P* (standing/sitting)		0.26		**0.000022**		**0.000022**		**0.049**	

*P* ^*^: Friedman's ANOVA.

*P*: Tukey's post hoc test.
